# Cross-Cultural Adaptation and Validation of the Chinese Version of the Brace Questionnaire

**DOI:** 10.3389/fped.2021.763811

**Published:** 2022-01-13

**Authors:** Honglei Yi, Hu Chen, Xinhui Wang, Hong Xia

**Affiliations:** ^1^Department of Orthopedics, People's Liberation Army General Hospital of Southern Theater Command, Guangzhou, China; ^2^Department of Orthopedics, The First School of Clinical Medicine, Southern Medical University, Guangzhou, China; ^3^Department of Anesthesiology, People's Liberation Army General Hospital of Southern Theater Command, Guangzhou, China

**Keywords:** adolescent idiopathic scoliosis (AIS), Brace Questionnaire, Chinese version, reliability, validity, SRS-22

## Abstract

**Objective:** To adapt the questionnaire cross-culturally and to analyze the adaptation and validation of the Chinese version of the Brace Questionnaire (C-BrQ).

**Methods:** The adaptation was based on the International Quality of Life Assessment Project guidelines. A total of 79 patients with AIS were included to examine the psychometric properties of the C-BrQ. The reliability was assessed using internal consistency (the Cronbach's alpha coefficient) and test–retest reliability (intraclass correlation coefficient ICC_2.1_, 95% CI). Floor and ceiling effects were calculated. Lin's concordance correlation coefficient (CCC, 95% CI) was used to compare the agreement between the Scoliosis Research Society-22 patient questionnaire (SRS-22) and C-BrQ.

**Results:** There were strong correlations between each item and its corresponding domain significantly. The correlations between the C-BrQ domains and their related questions vary from moderate to strong (*r* = 0.311–0.933, *P* < 0.05). The Cronbach's was 0.891, showing good internal consistency of each domain of the BrQ, and the ICC in test–retest was 0.860 (0.8776, 0.912), which means an excellent test–retest reliability. The Lin's CCC between SRS-22 and C-BrQ was 0.773 (0.669, 0.848), showing great agreement. However, no significant floor and ceiling effects in C-BrQ was observed except the ceiling effect in school activity and bodily pain.

**Conclusion:** BrQ was translated and cross-culturally adapted for use in China with good internal consistency and excellent test–retest reliability.

## Introduction

Adolescent idiopathic scoliosis (AIS) is traditionally defined as a lateral curvature of the spine on a frontal plane of 10° or more in patients aged 10–18 years of which the etiology is poorly understood. It is the most common type of scoliosis and frequently occurs in females (1–3). Previous studies dating from 1985 to 2011 showed that the prevalence of AIS ranges from 0.5 to 5.2% (3–5).

Most AIS patients do not need surgery. Brace therapy is considered to be the only effective way of conservative treatment. Based on Scoliosis Research Society (SRS) protocol, optimal inclusion criteria for AIS brace studies are listed as follows: (1) age is 10 or over when the brace is prescribed; (2) Risser's sign below grade 3; (3) primary curve angles between 25° and 40°; (4) no other prior treatment; (5) if female, either premenarchal or <1 year postmenarchal (6). In China, the indications of brace treatment are usually broad because it is a non-invasive way of treatment. Furthermore, the other potential causes are the heavy economic burden of patients' families and an unsatisfactory health insurance policy. As a result, for the immature children with Risser's sign 3, curve angles below 50° are often considered to be treated using the brace, but the wearing time can be adjusted accordingly.

High-correction bracing is shown to have favorable outcomes (7). However, bracing is considered to be an unpleasant experience that may affect self and body image, interpersonal communication, reducing overall quality of life (QoL) for patients eventually, especially for adolescents. What is more, wearing a brace for a long time may be harmful to the pressure areas of the body. Psychological problems and body pain have been the cause for AIS patients unlikely to accept brace treatment (8, 9). Therefore, doctors need not only to focus on the changes of the Cobb angles but also to pay more attention to the psychosocial functioning, which directly influence the benefit that patients receive from brace treatment (10).

The SRS-22 patient questionnaire is usually used to assess the QoL of idiopathic scoliosis (11). However, many qualitative factors related to conservative bracing therapy were not taken into consideration in the SRS-22. Developed by Botens–Helmus, another commonly used questionnaire called the Bad Sobernheim Stress Questionnaire (12) can only be used to evaluate the stress level of AIS patients caused by a brace. It is not child-specific and has no family-, school-, or activity-related problems involved. Created by Vasiliadis et al. in 2006, the Brace Questionnaire (BrQ) is the first questionnaire that specifically evaluates the QoL of AIS patients undergoing brace treatment (13). The questionnaire has already been adapted and translated into English, Korean (14), Persian (15), Italian (16), French (17), Polish (18), and Turkish (19) versions and languages without Chinese version. Thus, this study aims to adapt the questionnaire cross-culturally and to analyze the adaptation and validation of the Chinese version.

## Materials and Methods

### Participants

To examine the psychometric properties and clinical application of the adapted Chinese Brace Questionnaire, a survey was conducted in a group of 79 randomly selected outpatients. Ethical approval was received from the research committee of the author's institution. All the patients included were diagnosed with AIS by attending doctors.

Inclusion criteria were listed as follows: (1) 10–20 years of age; (2) brace treatment more than 2 months, at least 12 h per day; (3) Cobb angled from 20° to 50°, Risser sign: 0-III; (4) major thoracic, double major, thoracolumbar/lumbar scoliosis. Exclusion criteria included congenital scoliosis, neuromuscular scoliosis, or a history of prior spine treatment.

All patients filled in the C-BrQ and a previously validated Chinese (Mainland) version of the SRS-22 in the hospital with a consent form. Seven to 12 days after their first survey, most of the patients were asked to complete the tests again by phone or e-mail.

### Measurements

#### C-BrQ Questionnaire

The BrQ comprises 34 questions organized into eight domains: general health perception (questions 1 and 2), physical functioning (questions 3–9), emotional functioning (questions 10–14), self-esteem and aesthetics (questions 15 and 16), vitality (questions 17 and 18), school activity (questions 19–21), bodily pain (questions 22–27), and social functioning (questions 28–34). For questions 4–6, 12, and 14–17, “always” receives a score of 5, “most of the time” receives a score of 4, “sometimes” receives a score of 3, “almost never” receives a score of 2, and “never” receives a score of 1. For other questions, scoring rules are reversed. “Never” receives a score of 5 and “always” receives a score of 1. Each item score is then multiplied by 20, and the total score is divided by 34. The overall score ranges from 20 to 100. A higher score indicates better quality of life. A subscale score can be calculated for each of the eight domains by dividing the total score of each dimension by the number of questions it comprises.

According to international guidelines recommended by Beaton et al. (20), the process of intercultural adaptation of the C-BrQ bears a resemblance to our previous studies (21, 22). The original Greek Brace Questionnaire was translated into C-BrQ forward by two bilingual translators and one native Chinese-speaking spine surgeon independently. Next, discrepancies were found by comparing the three versions of translation carefully and reconciled by consensus. Being blinded to the study purpose, two independent native Greek translators whose second language was Chinese performed the backward translation. Neither of translators had a medical background or were unaware of the prior translation procedures. Each Greek translation was compared with the original Greek Brace Questionnaire and checked for inconsistencies by the translation team. Then, the team consolidated the original questionnaire, translations, back translations, and corresponding written reports to reach a consensus. The team discussed all the findings to develop the final C-BrQ, which was subjected to further psychometric testing.

#### SRS-22 Questionnaire

Developed by Haher et al. (23), SRS-22 is a practical and simple questionnaire to assess the quality of life of patients with idiopathic scoliosis. The questionnaire has been successfully adapted into a Chinese version with excellent validity and reliability (24), which comprises 22 questions, including parameters of function activity level, pain, mental health, self-image, and management satisfaction. Each question is scored from “1” to “5,” and in each domain, the recipient can score from 5 to 25 points except for the satisfaction from treatment subscale on which they can score from 2 to 10 points. The overall score ranges from 22 to 110 points. The higher the score, the better the quality of life. In addition, it is the mean values in each domain that are analyzed.

### Statistical Analysis

All data were analyzed by SPSS 20 (SPSS Inc., Chicago IL). Ordinal variables were presented as median and continuous variables as mean ± standard deviation. The floor and ceiling effect was defined as the proportion of patients included in the bottom 15% and top 15% in the range of the score, respectively. For each score, the percentage of patients in the floor or ceiling brackets was calculated and considered significant when >15% (17).

The internal consistency was assessed using internal consistency (the Cronbach's alpha coefficient), test–retest reliability was calculated using relative (intraclass correlation coefficient ICC_2.1_, CI = 95%) and absolute (standard error of measurement, SEM, and minimum detectable change, MDC) estimates. The second model of ICC (two-way-mixed) was used in this study for the reason that the within-column and between-columns effects were random as well. In addition, the intended measurement was based on the same person (single measurement), so type 1 was used. SEM, as an indication of expected measurement error in a single individual's score using the same units as the points, was calculated as SD${}^{*}\sqrt{1-ICC}$. MDC was calculated at the 90% level, which is appropriate for assessing change for routine clinical use. MDC = SEM${}^{*}{\sqrt{2}}^{*}$1.64. It was used to provide the threshold amount of change in scores required for the rater to be 90% confident that true change beyond that of measurement error had occurred. In addition, Lin's CCC (95% CI) was used to compare the agreement between the SRS-22 patient questionnaire (SRS-22) and C-BrQ. The difference was considered statistically significant when *P* < 0.05.

## Results

Table 1 presents the characteristics of subjects, a total of 79 AIS patients (11 male) were investigated from March 2018 to February 2019. Seven patients did not answer the phone or reply to the email to complete the second questionnaire. All of the patients completed the C-BrQ Questionnaire within 15 min. Table 2 shows the distribution of the results for the five domains of the SRS-22 questionnaire, including the mean values, number of items, and standard deviations for each domain. Table 3 describes basic data of each C-BrQ domain and the percentage of subjects scoring minimum (floor effect) and maximum (ceiling effect). There was no significant floor and ceiling effects in C-BrQ except a ceiling effect in school activity and bodily pain.

**Table 1:** The demographic characteristics of the study population.

	**Means (SD)**
Population (*N*)	79
Gender (*N*)	11
Age (years)	14.17 (2.00)
Cobb angle of curve (°)	32.23 (4.25)
Types of curve (*N*)	
Major thoracic	35
Double major	13
Thoracolumbar/lumbar scoliosis	31
Duration of brace wearing (total, months)	16.98 (20.59)
Brace compliance (per day, hours)	18.12 (4.49)

**Table 2:** Basic data of each SRS-22 domain.

**SRS-22 domain**	**Number of items**	**Mean (SD)**
Function	5	4.18 (0.57)
Pain	5	4.32 (0.50)
Self-image	5	3.46 (0.65)
Mental health	5	3.88 (0.73)
Management	2	3.80 (0.80)
Total	22	3.95 (0.49)

**Table 3:** Basic data of each C-BrQ domain.

**BrQ domain**	**Number of items**	**Median**	**Mean ± SD**	**Number (%)**	
				**Floor effects**	**Ceiling effects**
Total	34	77.64	77.10 ± 9.61	0 (0%)	0 (0%)
General health perception	2	3.00	3.16 ± 0.905	2 (2.5%)	4 (5.1%)
Physical functional	7	3.85	3.82 ± 0.718	2 (2.5%)	2 (2.5%)
Emotional functional	5	3.60	3.60 ± 0.725	0 (0%)	2 (2.5%)
Self-esteem and aesthetics	2	3.00	2.72 ± 1.012	10 (12.7%)	3 (3.8%)
Vitality	2	3.50	3.31 ± 0.935	3 (3.8%)	4 (5.1%)
School activity	3	4.33	4.33 ± 0.659	0 (0%)	27 (34.2%)
Bodily pain	6	4.50	4.39 ± 0.620	0 (0%)	31 (39.2%)
Social functional	7	4.14	4.07 ± 0.674	0 (0%)	11 (13.9%)

### Reliability

Table 4 shows the internal consistency of C-BrQ. There were strong correlations between each item and its corresponding domain significantly. The correlations between the C-BrQ domains and their related questions vary from moderate to strong (*r* = 0.311–0.933). In addition, it showed the very good internal consistency of the BrQ (Cronbach's *a*, 0.891) and each domain (Cronbach's *a*, 0.659~0.850).

**Table 4:** Internal consistency.

**Domains and questions**	**Mean ± SD**	**Cronbach's**	**Pearson**	***p***
		**alpha**	**correlation**	
Total	77.10 ± 9.608	0.891		
General health perception	3.16 ± 0.905	0.739		
1	3.23 ± 0.831		0.861	0.000
2	3.10 ± 1.172		0.933	0.000
Physical functional	3.82 ± 0.718	0.793		
3	3.66 ± 0.932		0.677	0.000
4	3.22 ± 1.021		0.405	0.000
5	3.87 ± 1.244		0.770	0.000
6	3.87 ± 1.265		0.746	0.000
7	4.00 ± 1.050		0.666	0.000
8	4.06 ± 1.017		0.670	0.000
9	4.08 ± 0.944		0.741	0.000
Emotional functional	3.60 ± 0.725	0.668		
10	3.73 ± 0.996		0.773	0.000
11	3.81 ± 0.948		0.700	0.000
12	3.52 ± 1.175		0.670	0.000
13	2.94 ± 1.371		0.598	0.000
14	3.99 ± 0.980		0.595	0.000
Self-esteem and aesthetics	2.72 ± 1.012	0.812		
15	2.71 ± 1.100		0.917	0.000
16	2.73 ± 1.106		0.918	0.000
Vitality	3.31 ± 0.935	0.677		
17	3.22 ± 1.009		0.853	0.000
18	3.41 ± 1.138		0.887	0.000
School activity	4.33 ± 0.659	0.659		
19	4.08 ± 0.971		0.821	0.000
20	4.78 ± 0.570		0.571	0.000
21	4.15 ± 0.962		0.889	0.000
Bodily pain	4.39 ± 0.620	0.850		
22	4.92 ± 0.267		0.311	0.005
23	4.22 ± 0.915		0.786	0.000
24	4.25 ± 0.899		0.870	0.000
25	4.19 ± 0.935		0.864	0.000
26	4.41 ± 0.809		0.875	0.000
27	4.37 ± 0.894		0.700	0.000
Social functional	4.07 ± 0.674	0.705		
28	4.23 ± 1.120		0.522	0.000
29	3.81 ± 1.199		0.397	0.000
30	3.78 ± 1.140		0.795	0.000
31	4.59 ± 0.651		0.563	0.000
32	4.00 ± 1.240		0.651	0.000
33	4.39 ± 0.953		0.652	0.000
34	3.75 ± 1.319		0.686	0.000

Table 5 shows that the test–retest study revealed high reliability, and the value of ICC_2.1_ for the total score was high (0.860, CI ranged from 0.877 to 0.912) with SEM = 0.494 and MDC 90% CI = 0.573.

**Table 5:** Test–retest reliability indicators.

**Domain**	**ICC (95% CI)**	**SEM**	**MDC^†^**
Total	0.860 (0.877, 0.912)	0.494	0.573
General health perception	0.917 (0.868, 0.948)	0.032	0.036
Physical functional	0.930 (0.889, 0.956)	0.023	0.027
Emotional functional	0.882 (0.812, 0.926)	0.030	0.035
Self-esteem and aesthetics	0.831 (0.731, 0.895)	0.053	0.062
Vitality	0.856 (0.770, 0.910)	0.044	0.051
School activity	0.819 (0.711, 0.887)	0.031	0.036
Bodily pain	0.809 (0.694, 0.880)	0.029	0.034
Social functional	0.923 (0.876, 0.952)	0.023	0.026

*ICC, intraclass correlation coefficient; CI, confidence interval; SEM, standard error of measurement; MDC, minimum detectable change*.

† *At the 90% confidence level*.

### Validity

Table 6 shows the analysis of the correlation between C-BrQ and SRS-22 scores. There is a strong correlation between total C-BrQ and SRS-22 (CCC: 0.773, 95% CI: 0.669, 0.848). Also, C-BrQ domain correlated with the single domain scores of SRS-22 (CCC range from 0.307 to 0.574).

**Table 6:** LIN's CCC (95% CI) between the BrQ scores and the SRS-22.

	**SRS-22**	**SRS-22**	**SRS-22**	**SRS-22**	**SRS-22**	**SRS-22 Management**
	**total**	**function**	**pain**	**self-image**	**Mental health**	**satisfaction**
BrQ-Total	0.773 (0.669, 0.848)	0.525 (0.371, 0.650)	0.435 (0.284, 0.564)	0.513 (0.367, 0.635)	0.574 (0.429, 0.690)	0.307 (0.122, 0.470)

*LIN's CCC, Lin's Concordance Correlation Coefficient*.

## Discussion

Currently, a long period of brace wearing is often recommended to treat AIS patients as a conservative treatment (7). Considerable attention should be paid to patients' HRQoL variables besides the radiological changes because wearing a brace negatively affects body image, causes pain, and creates pressure discomfort, which may decrease the QoL and efficacy of conservative treatment as well (8–10). SRS-22, the short form (36) health survey, and the Bad Sobernheim Stress Questionnaire are available, but they do not include any specific question on brace therapy and how it affects QoL (12, 24, 25). The BrQ is the first questionnaire that specifically evaluates the QoL of AIS patients undergoing brace treatment including family-, school-, and activity-related problems (13). It is widely used all over the world. However, the Chinese version of the Brace Questionnaire has not been reported yet.

In this study, the questionnaire is translated, back-translated, modified, and predicted based on the guidelines by Beaton et al. (20). To respect the expression of the original scale, culturally relevant modifications are made to the word choice, syntactical construction, and ambiguous terms in accordance with Chinese tradition. ICC is the most commonly applied statistical parameter for showing the internal consistency of an instrument. Cronbach's alpha is the most commonly applied statistical parameter for showing the internal consistency of an instrument. Generally speaking, once the coefficient is more than 0.8, the internal consistency of an instrument is considered to be satisfactory (26). In this study, the Cronbach's alpha coefficient for the C-BrQ was 0.891, showing that this translated version is reliable. That is in agreement with the values reported by others. The Cronbach's α value for the original Greek version is 0.82 (13), for the French version (14) 0.85, for the Turkish version (19) 0.94, for the Polish version (18) 0.94, for the Italian version (16) 0.86, for the Korean version (14) 0.872, and for the Persian version (15) 0.79. The ICC of this study was 0.86, which indicated excellent reliability and were the same as reported by others: 0.96 (Persian) (15), 0.913 (Korean) (14), 0.943 (Italian) (16), 0.95 (Turkish) (19). No definitive time interval has been experimentally determined, and 2 days−2 weeks are often considered as a time period to evaluate test–retest reliability (27). All of these test–retest studies were made 7–12 days after the first questionnaires because the outpatients cannot wait for a longer time to complete the first and second questionnaires while visiting the clinic. Therefore, we use telephone or email to connect the patients at home.

In the analysis for convergent validity, our study shows a strong relationship exists between the total C-BrQ and SRS-22 scores (CCC: 0.773, 95% CI: 0.669, 0.848), indicating the high validity of the questionnaires. This relationship is also found in the other studies with *r* = 0.71 in Persian (15), 0.64 in Turkish (19), 0.712 in Korean (14), 0.826 in Italian (16), respectively.

Although the findings from this study provide strong support for the validity and reliability of the Chinese version of BrQ, there are still some limitations of this study. First, the patients included had mild to moderate scoliosis with a mean Cobb angle of around 32° (22°–42°). However, some studies report that moderate-to-severe scoliosis (e.g., 45°–60°) in adolescents still accepted brace treatment with good results (28, 29). Whether C-BrQ could be applied in patients with these types of scoliosis need to explore further. Second, all of the participants used the Boston brace instead of the Milwaukee brace, which was used in the aforementioned studies. Different type of rigid brace may have different negative effects on the HRQoL in patients with AIS, which may be the risk of bias (30, 31).

## Conclusions

BrQ was translated and cross-culturally adapted for use in China with good internal consistency and excellent test–retest reliability. We suggest that the C-BrQ can be widely used for assessing the HRQoL of adolescents with idiopathic scoliosis undergoing bracing treatment in mainland China.

## Data Availability Statement

The raw data supporting the conclusions of this article will be made available by the authors, without undue reservation.

## Ethics Statement

The studies involving human participants were reviewed and approved by the Institutional Review Committee of People's Liberation Army (PLA) General Hospital of Southern Theatre Command. Written informed consent to participate in this study was provided by the participants' legal guardian/next of kin.

## Author Contributions

All authors contributed to the questionnaire and data collection. The first draft of the manuscript was written by HY and HC. Data collection and analysis were performed by XW and HX. All authors read and approved the final manuscript.

## Funding

This research was supported by the National Natural Science Foundation of China (81972080), the Science and Technology Planning Project of Guangdong Province (2017B030314139), and the Natural Science Foundation of Guangdong Province (2015A030312004).

## Conflict of Interest

The authors declare that the research was conducted in the absence of any commercial or financial relationships that could be construed as a potential conflict of interest.

## Publisher's Note

All claims expressed in this article are solely those of the authors and do not necessarily represent those of their affiliated organizations, or those of the publisher, the editors and the reviewers. Any product that may be evaluated in this article, or claim that may be made by its manufacturer, is not guaranteed or endorsed by the publisher.
